# Integrated Analyses of m6A Regulator-Based Signature on Its Clinical Application and Immunogenomic Landscape in Stomach Adenocarcinoma

**DOI:** 10.1155/2022/2053719

**Published:** 2022-09-20

**Authors:** Lan Zhang, Qing Zhang, Zebo Huang, Mingxia Zhu, Tongshan Wang, Wei Zhu, Xiaping Wang, Xin Zhou

**Affiliations:** ^1^Department of Radiation Oncology, Shanghai Tenth People's Hospital of Tongji University, Shanghai, China; ^2^Department of Neurosurgery, Xinghua People's Hospital, Xinghua, 225700 Jiangsu, China; ^3^Department of Oncology, The Affiliated Hospital of Jiangnan University, Wuxi 214062, China; ^4^Department of Radiation Oncology, The First Affiliated Hospital of Soochow University, Suzhou 215006, China; ^5^Department of Oncology, First Affiliated Hospital of Nanjing Medical University, Nanjing 210029, China; ^6^Department of Pathology, The Second Affiliated Hospital of Nanjing Medical University, Nanjing 210000, China

## Abstract

**Background:**

The whole tumor microenvironment (TME) infiltration features monitored by integrated roles of different RNA N6-methyladenosine (m6A) regulators remain elusive. Our study is aimed at exploring the association between m6A modification patterns, TME cell-infiltrating levels, and patients' prognosis in stomach adenocarcinoma (STAD) patients.

**Methods:**

Consensus clustering was performed based on the integrated analyses of 17 m6A regulators and 229 m6A-related hallmark genes in STAD (The Cancer Genome Atlas (TCGA) cohort, *n* = 443; Gene Expression Omnibus (GEO) GSE57303, *n* = 70, GSE62254 *n* = 300, and GSE84437 *n* = 433). A m6ASig scoring system was calculated by the principal component analysis (PCA), and its prognostic value was validated in an independent dataset GES15459.

**Results:**

Three m6A clusters were identified among 1246 STAD patients, which had significant overall survival (OS) differences and demonstrated different TME immune cell infiltration and biological behaviors. According to the m6ASig score, which was generated from the m6A-related hallmark genes, STAD patients were divided into the high-m6ASig group (*n* = 585) and low-m6ASig group (*n* = 586). Patients in the high-m6ASig group had a notably prolonged OS and higher immune cell infiltration. Moreover, patients with higher m6ASig score were associated with higher microsatellite instability (MSI); higher PD-L1, CTLA4, and ERBB2 expressions; and greater tumor mutation burden (TMB). Patients with higher m6ASig score demonstrated a better immune response and drug sensitivity.

**Conclusion:**

Our m6ASig scoring system could characterize TME immune cell infiltration, thus predict patient's prognosis and immunotherapy and chemotherapy efficacy, offering a novel tool for the individualized therapeutic implications for STAD patients.

## 1. Introduction

Gastric cancer (GC) is one of the most fatal malignancies worldwide and ranks second in tumor-related death in China [1]. STAD (stomach adenocarcinoma) accounts for the vast majority of gastric cancer [2]. Most of STAD patients are diagnosed at advanced stages and lost opportunity for surgery. Chemotherapy remains the most classic way for STAD treatment [3]. However, patients with the same stage and similar treatment regimens demonstrated large variations in clinical outcomes [4]. Recently, monoclonal antibodies targeting cytotoxic T lymphocyte-associated antigen 4 (CTLA4), programmed death-1 (PD-1) receptor, and its ligand (PD-L1) have achieved encouraging antitumor effects in STAD patients [5]. However, only a limited number of patients demonstrated durable responses from current immunotherapies [5]. Thus, new strategies are urgently warranted for improvement of prognostic prediction and therapy strategy.

Methylation of N6 adenosine (m6A) is the most abundant and predominant modification of mRNA, lncRNAs, and miRNAs detected in higher mammalian cells [6]. An average of 1000 nucleotides are observed to have 1-4 m6A adenosine residues [7]. m6A modification process is dynamic and reversible, which is regulated by three types of regulatory enzymes: “writers” (methyltransferases), “readers” (binding proteins), and “erasers” (demethylases) [7]. Studies of those m6A modification regulators have helped to depict the exact functions of m6A methylation in posttranscriptional level. Emerging literature has reported that the m6A regulators could mediate gene expression levels in diverse biological processes, including cancer development, progression invasion, and metastasis, and could serve as prognostic biomarkers [8–11]. Moreover, m6A regulator expression is tightly associated with the tumor immune microenvironment (TME) [6]. The TME components such as cytotoxic T cells, helper T cells, dendritic cells (DCs), and tumor-associated macrophages (TAMs) could reflect the immune response [12] and chemotherapy benefit [13]. Therefore, by comprehensively parsing the m6A modification and TME relationships, novel therapeutic biomarkers are likely to be revealed. In fact, several studies have reported the interactions between m6A modification and TME immune cell infiltrations in STAD [14–16]. However, most of the studies focused on limited number of m6A regulators, while the antitumor effect is mediated cooperatively by different tumor-associated factors.

Hence, through bioinformatics analysis from both the TCGA and GEO databases, we comprehensively analyzed the association between m6A modification patterns, TME cell-infiltrating levels, and patients' prognosis in STAD. Moreover, we constructed an m6ASig scoring system to quantify the m6A modification and predict patients' clinical responses to immunotherapy and chemotherapy in individual patients.

## 2. Materials and Methods

### 2.1. Data Collection and Preprocessing

The flowchart of the present study was shown in Figure S1. Raw counts and fragments per kilobase of transcript per million- (FPKM-) normalized RNA-seq data, single nucleotide variation (SNV), and copy number variation (CNV) data with corresponding clinical information of STAD patients were downloaded from The Cancer Genome Atlas (TCGA) database (https://portal.gdc.cancer.gov/) on July 29, 2021. The count data of genes in each sample were transformed to transcripts per million (TPM) values for normalization. A total of 443 patients with full clinical information, including age, gender, T stage, N stage, M stage (according to the AJCC 8th), and survival data, were included. In addition, three eligible datasets GSE57303 (70 patients), GSE62254 (300 patients), and GSE84437 (433 patients) containing mRNA microarray data and prognostic information were retrieved from Gene Expression Omnibus (GEO) database. The clinical characteristics of the TCGA and GEO patients are summarized in Table 1. An independent dataset GES15459, which lacked clinic data of T, N, and M stages, was selected as an external validation cohort to verify the prognostic role of the m6ASig model (Table S1). The R language software (v3.6.3) and relevant packages were used in the process of data analyses in the current study. And a two-sided *P* value < 0.05 was defined as statistical significance unless specifically indicated.

### 2.2. m6A RNA Methylation Regulator Detection and Prognosis Analysis

Twenty-three well-acknowledged m6A RNA methylation regulators, including 8 “writers” (METTL3, METTL14, METTL16, WTAP, VIRMA, ZC3H13, RBM15, and RBM15B), 13 “readers” (YTHDC1-2, YTHDF1-3, HNRNPC, FMR1, LRPPRC, HNRNPA2B1, IGFBP1-3, and RBMX), and 2 “erasers” (FTO and ALKBH5), were collected according to previous studies [17, 18]. The differential expression patterns of the 23 m6A regulators were analyzed with the R “limma” package in the TCGA cohort. We visualized the chromosome location and mutational landscape of the 23 regulators using the “RCircos” and “maftools” packages, respectively. Later, STAD patients in TCGA cohort and three GEO cohorts were consolidated as a STAD metacohort. The batch effects were removed by the “combat” function of the “sva” package. The patients in STAD metacohort were classified into high- or low-expression groups according to the cut-off value of each gene acquired with the function “surv_cutpoint” of the “survminer” package. The differences in overall survival (OS) were evaluated via “survival” and “survminer” packages. Interactions among m6A regulators were analyzed, and a network was depicted by “igraph, psych, reshape2, and RColorBrewer” packages.

### 2.3. Consensus Clustering and PCA Analysis

To functionally illustrate the biological role of m6A regulators in STAD, patients in STAD metacohort were clustered into different groups using “ConsensusClusterPlus” package. Principal component analysis (PCA) is an unsupervised statistical method, which is able to condense data sources down into a group of linearly uncorrelated variables [19]. Hence, PCA was performed using “PCA, limma, and ggplot” packages to observe the distribution of m6A regulator expressions in different clusters. K-M survival curves were utilized to assess the survival differences between clusters. The distribution of different clinicopathological characteristics between the three clusters were visualized by “pheatmap.”

### 2.4. Gene Set Variation Analysis

The ssGSEA (single-sample gene set enrichment analysis) algorithm was applied to identify the relative abundance of infiltrating immune cells related to different clusters (including activated B cells, activated CD4+ cells, activated CD8 + T cells, activated dendritic cells, CD56 bright natural killer cell, CD56dim natural killer cell, eosinophil, gamma delta T cell, immature B cell, immature dendritic cell, MDSC, macrophage, mast cell, monocyte, natural killer T cell, natural killer cell, neutrophil, plasmacytoid dendritic cell, regulatory T cell, T follicular helper cell, type 1 T helper cell, type 17 T helper cell, and type 2 T helper cell) in the tumor microenvironment. “Reshape2, ggpubr, limma, GSEABas, and GSVA” algorithms were used to calculate and depict the immune infiltration of different m6A clusters. Gene set variation analysis (GSVA) [20] was performed to find the most significant pathways between different m6A regulator clusters, according to the gene sets “c2.cp.kegg.v7.4.symbols.gmt.”

### 2.5. Screening of m6A-Related Genes Affecting Prognosis

The differentially expressed genes (DEGs) in the three m6A clusters were obtained by setting a threshold *P* < 0.01. Cox regression analysis was used to identify the m6A cluster hallmark genes. Finally, a total of 229 DEGs were obtained. Next, consensus clustering and PCA analysis were again performed to determine subgroups of STAD patients based on the expression similarity of m6A cluster hallmark genes.

### 2.6. Gene Ontology and Kyoto Encyclopedia of Genes and Genomes Analyses

To explore the relevant biological pathways enriched by these DEGs, “clusterProfiler, org.Hs.eg.db, enrichplot, and ggplot2” packages were applied to analyze and visualize Gene Ontology (GO) and Kyoto Encyclopedia of Genes and Genomes (KEGG) enrichment paths.

### 2.7. Construction and Evaluation of the m6ASig Model

Additionally, an m6ASig model was constructed with the combination of PC1 and PC2 from PCA analysis of m6A cluster hallmark genes: m6ASig = PC1 + PC2. Patients were classified into two different groups according to the median value of the m6ASig score followed by Kaplan-Meier analyses. Next, our study created a Sankey diagram to illustrate the patient transitions between m6A clusters, m6A cluster hallmark gene clusters, m6ASig, and their living status. Univariate and multivariate cox regression analyses involving all variables were carried out to explore the independent prognostic elements for STAD patients. Nomogram is an effective predictive tool that is able to generate an individual probability of a clinical event [21]. Hence, we constructed a predictive nomogram by integrating different prognostic factors using the “survival and regplot” and “rms” R packages.

### 2.8. Association between Different m6ASig Score Groups and Tumor Immune Landscape

The association between different m6ASig score groups and the 23 types of infiltrating immune cells was analyzed using the “corrplot” algorithm. Microsatellite instability (MSI) is a molecular tumor phenotype of a deficient mismatch repair system [22]. MSI status of patients in TCGA cohort was retrieved from The Cancer Immunome Atlas (TCIA, https://tcia.at/home). Analysis regarding the association between m6ASig score and MSI in the TCGA cohort was achieved by “limma” package.

Programmed cell death protein 1 or programmed cell death ligand 1 (PD-1/PD-L1) is a checkpoint molecule that plays a vital role in the immune system suppression [23]. ErbB2 is an important signal integrator for the epidermal growth factor family of receptor tyrosine kinases [24]. Hence, the corrections between m6ASig score and PD-L1, CTLA-4, and ERBB2 were analyzed with the “limma” package.

Tumor mutational burden (TMB) is a measure of the amount of somatic coding mutations per DNA megabase [25]. TMB data of patients in TCGA database was calculated through the “maftools” package in R. Patients in TCGA cohort were divided into the low TMB group (*n* = 68) and the high TMB group (*n* = 291) according to cut-off value of 1 mutation/mb for further analysis. The correlation between TMB and m6ASig score was conducted by “ggpubr and reshape2” R packages. We also used the “survival” package to compare the survival time differences.

### 2.9. Chemotherapy and Immunotherapy Response Prediction

To assess the predictive role of the m6ASig model for chemotherapy in STAD patients, we used the “pRRophetic” package to predict the half-maximal inhibitory concentration (IC_50_) of some common chemotherapy drugs, including cisplatin, docetaxel, paclitaxel, and rapamycin. Next, based on The Cancer Immunome Atlas (TCIA) [26], we further explored the correlation between different m6ASig score groups and immunophenoscore (IPS), which act as a marker for immune response. The results were visualized by “ggpubr” R package.

### 2.10. Statistical Analysis

Statistics analysis was conducted on R platform v3.6.3. Mann–Whitney *U* tests were performed to compare m6A regulators and m6A-related gene expression levels in different subgroups (normal tissues, tumor tissues, and different clusters). Chi-squared test or Fisher's exact test was exerted to compare the clinical phenotypes between different clusters, as well as the immune responses between high-m6ASig score group and low-m6ASig score group. Pearson correlation analysis was conducted to assess the correlation between m6ASig score and different gene clusters. Univariate and multivariate cox regression analyses were utilized to determine the independent prognostic factors for STAD patients. Kaplan-Meier (K-M) curves and log-rank test were performed to compare the survival differences. All tests were two-tailed, and a *P* value of 0.05 or specifically specified was considered statistically significant.

## 3. Results

### 3.1. Expression Patterns of m6A RNA Methylation Regulators in STAD

**Figure 1:**
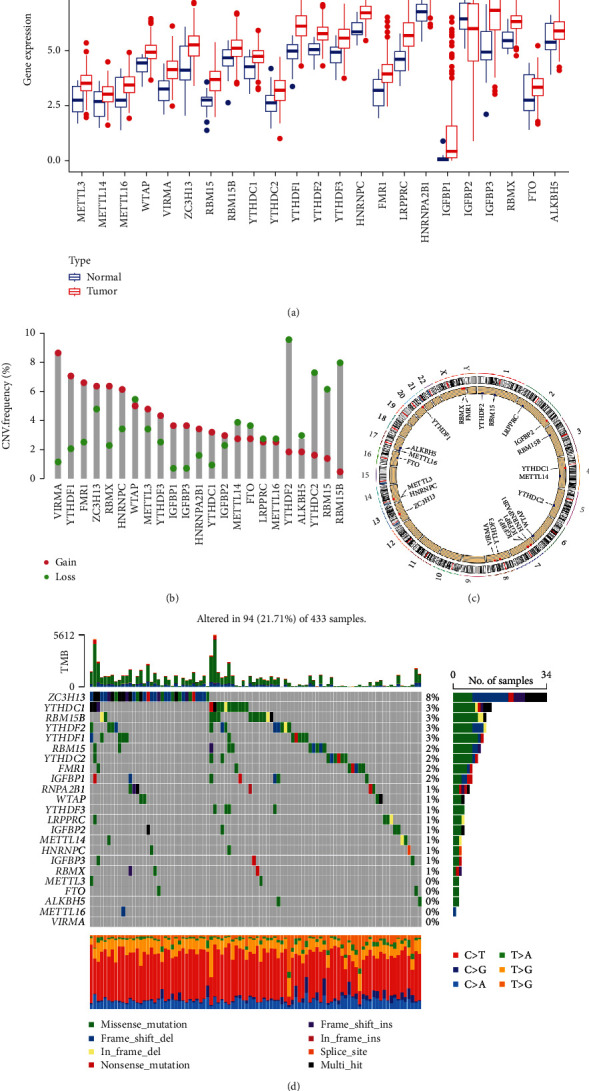
Overview of the 23 m6A regulators in stomach adenocarcinoma (STAD). (a) The mRNA expression of the m6A regulators in STAD tissues and normal tissues. (b) The CNV variation frequency of the m6A regulators. Red dots: CNV amplification; green dots: CNV deletion. (c) The location of CNV alteration of the m6A regulators on different chromosomes. (d) Mutation frequency of the m6A regulators of STAD patients in the TCGA cohort (^∗^*P* < 0.05, ^∗∗^*P* < 0.01, and ^∗∗∗^*P* < 0.001).

Our study firstly analyzed the expression levels of the 23 m6A regulators in STAD and adjacent normal tissues based on the TCGA cohort. As shown in Figure 1(a), 22 of the 23 m6A regulators had significantly higher expression in STAD tissues than normal ones, while the expression level of the “reader” IGF2BP2 was notably lower in STAD tissues.

### 3.2. Genetic Alteration of m6A Regulators in STAD

The CNV and SNV data of 23 m6A regulators were analyzed using TCGA data to assess the impacts of genetic changes on the gene expression. The results demonstrated that CNVs of the 23 m6A regulators could be frequently spotted. More specifically, the m6A “reader” gene YTHDF2 owned the highest frequency of CNV events, which mainly had decreased copy numbers in STAD tissues (Figure 1(b)). The chromosome location of the 23 m6A regulators is shown in Figure 1(c). Next, we explored the SNV mutations in the 23 m6A regulators. As shown in Figure 1(d), the m6A “writer” gene ZC3H13 had the highest frequency of genetic alteration (8%), and Frame Shift Del was the most frequent alteration.

### 3.3. Survival Analysis of m6A Regulators

**Figure 2:**
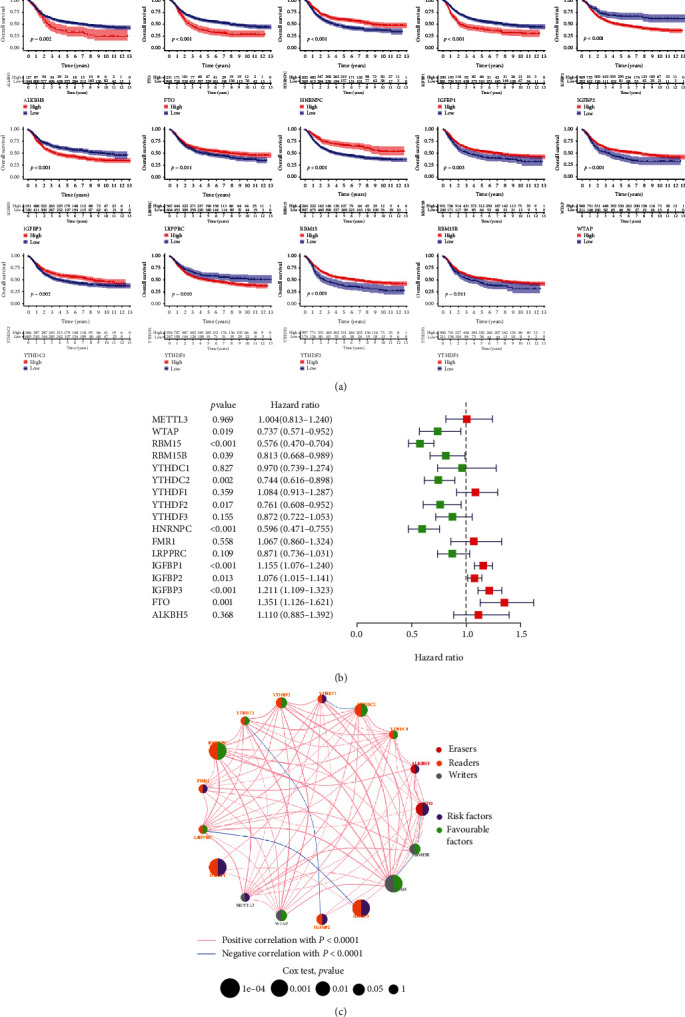
Overall survival (OS) analysis of 17 m6A regulators (METTL3, WTAP, RBM15, RBM15B, YTHDC1, YTHDC2, TYHDF1, YTHDF2, YTHDF3, HNRNPC, FMR1, LRPPRC, IGFBP1, IGFBP2, IGFBP3, FTO, and ALKBH5). (a) Kaplan-Meier survival curve for ALKBH5, FTO, HNRNPC, IGFBP1, IGFBP2, IGFBP3, LRPPRC, RBM15, RBM15B, WTAP, YTHDC2, TYHDF1, YTHDF2, and YTHDF3. (b) Cox regression analysis identified the independent prognostic predictors in the TCGA-GEO metacohort. (c) Interactions among different m6A regulators in the TCGA-GEO metacohort. *P* < 0.05 was considered significant.

A total of 17 m6A regulatory genes (METTL3, WTAP, RBM15, RBM15B, YTHDC1, YTHDC2, TYHDF1, YTHDF2, YTHDF3, HNRNPC, FMR1, LRPPRC, IGFBP1, IGFBP2, IGFBP3, FTO, and ALKBH5) were assessed and recruited for survival analysis in the metadata cohort by combining the TCGA and the three GEO datasets (GSE57303, GSE62254, and GSE84437). Kaplan-Meier curves showed that higher expression levels of ALKBH5, FTO, IGFBP1, IGFBP2, IGFBP3, and YTHDF1 were significantly associated with poorer OS in STAD patients (Figure 2(a)), while patients with lower expression levels of HNRNPC, LRPRPC, RBM15, RBM15B, WTAP, YTHDC2, YTHDF2, and YTHDF3 showed shortened OS (Figure 2(a)). In cox regression analysis, high expression levels of YTHDF1, FMR1, IGFBP1, IGFBP2, IGFBP3, FTO, and ALKBH5 were unfavorable factors for STAD patients' OS (Figure 2(b)), while patients with higher levels of WTAP, RBM15, YTHDC2, TYHDF2, YTHDF3, HNRNPC, and LRPPRC were connected with better OS (Figure 2(b)). In addition, correlations among the expression patterns of the 17 m6A regulators were further explored. Results demonstrated that the majority of the m6A regulators were positively connected with one another, expect for two connections: LRPPRC and IGFBP3 and YTHDF3 and IGFBP2 (Figure 2(c)).

### 3.4. Consensus Clustering of m6A Regulators with Different Clinical Characteristics and Survival

**Figure 3:**
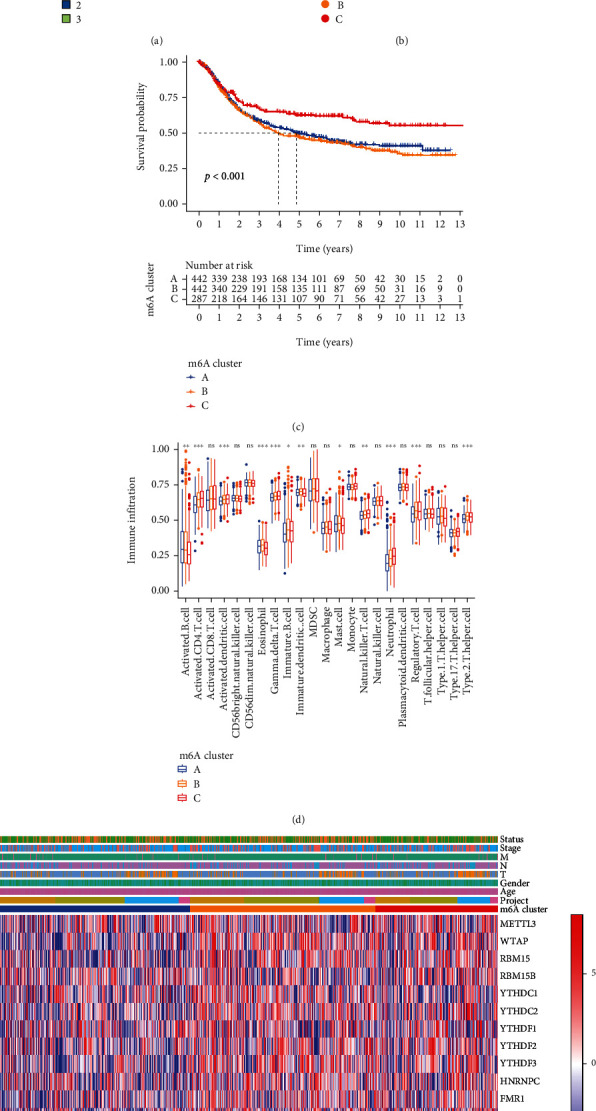
Consensus clustering based on the expression of 17 m6A regulators and the survival differences, distinct immune landscapes, and clinicopathologic characteristics of the three clusters. (a) The overlap among clusters when *k* = 3. (b) Scatter plot of principal component analysis (PCA) for m6A regulators in the TCGA-GEO metacohort. (c) Kaplan-Meier curves for cluster A, cluster B, and cluster C (log-rank test, *P* < 0.001). (d) TME cell infiltration characteristics in the three clusters in the TCGA-GEO metacohort (^∗^*P* < 0.05, ^∗∗^*P* < 0.01, and ^∗∗∗^*P* < 0.001). (e) Heatmap showing the relationship among the clinicopathological characteristics of the three clusters.

Since m6A regulatory genes are connected with STAD prognosis, consensus clustering and PCA analysis were performed according to the expression patterns of 17 m6A regulatory genes. When the clustering stability ranging from 2 to 9, *k* = 3 was proved to be the optimal point to acquire the biggest differences between different clusters. A total of 1171 STAD patients from both TCGA and GEO cohorts were clustered into three subtypes: cluster A (*n* = 442), cluster B (*n* = 442), and cluster C (*n* = 287) (Figures 3(a) and 3(b)). As shown in Figure 3(c), the overall survival (OS) of cluster C was significantly longer than other clusters (*P* < 0.001). Next, we analyzed the immune infiltration levels in different clusters. Cluster A had higher infiltration levels of activated B cell, and cluster B had higher infiltration levels of eosinophil, immature B cell, immature dendritic cell, MDSC, mast cell, regulatory T cells (Tregs), and type 2 T helper cell, whereas cluster C was more associated with activated CD4 T cells, activated dendritic cells, gamma delta T cells, natural killer cells, and neutrophil infiltration (Figure 3(d)). The comprehensive landscapes of correlations between the different clusters and clinicopathological characteristics were visualized in the form of heatmaps (Figure 3(e)). Next, GSVA was performed to assess the potential mechanisms leading to the differences in the immune infiltration among the three subgroups. Compared with cluster B, cluster C was mainly involved in base excision repair and homologous recombination pathways (Figure S2).

### 3.5. Consensus Clustering of m6A-Related Gene Cluster

**Figure 4:**
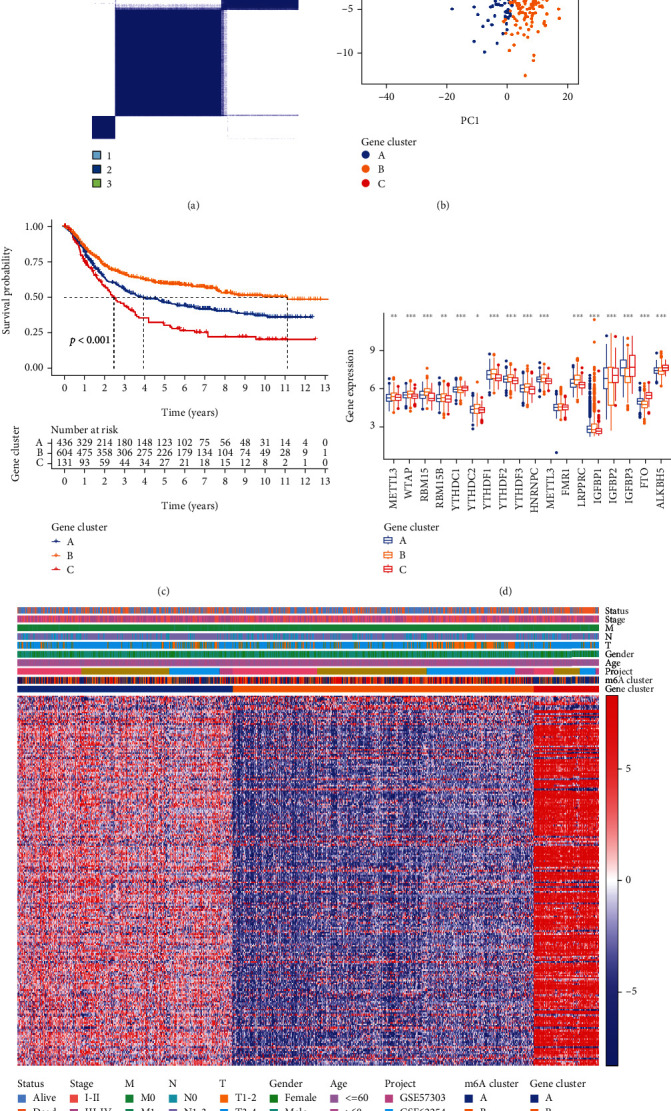
Consensus clustering based on the 229 m6A hallmark genes and the survival differences, distinct immune landscapes, and clinicopathologic characteristics of the three clusters. (a) The overlap among clusters when *k* = 3. (b) Scatter plot of principal component analysis (PCA) of m6A hallmark genes in the TCGA-GEO meta-cohort. (c) Kaplan-Meier curves for cluster A, cluster B, and cluster C (log-rank test, *P* < 0.001). (d) The gene expression patterns of 17 m6A regulators among the three clusters (^∗^*P* < 0.05, ^∗∗^*P* < 0.01, and ^∗∗∗^*P* < 0.001). (e) Heatmap showing the relationship among the clinicopathological characteristics of the three clusters.

Next, we investigated whether there was a persistent dysregulation of certain genes among the three m6A clusters. A total of 292 DEGs were identified through comparing the expression patterns of mRNAs across the three clusters (Table S2, Figure S3A). Then, 229 genes were identified as m6A hallmark genes via cox regression analysis (Table S3). GO and KEGG enrichment analyses demonstrated that those DEGs were mainly involved in the signal pathways related to cancer development and metastasis, such as transcriptional misregulation and Wnt signaling pathway (Figure S3B, C). To examine the clinical significance of the DEGs, STAD samples were classified into 3 clusters using the ConsensusClusterPlus tool (Figures 4(a) and 4(b)). Prognostic analysis revealed that cluster B had a better OS than clusters A and C (*P* < 0.001). In addition, we explored the m6A regulator expression in different gene clusters. METTL3, WTAP, RBM15, RBM15B, YTHDC2, YTHDF1, YTHDF2, YTHDF3, HNRNPC, FMR1, LRPPRC, and IGFBP1 expressions were significantly higher in cluster B, while YTHDC1, IGFBP2, IGFBP3, FTO, and ALKBH5 had lower expression levels in cluster B.  Figure 4(e) depicts the heatmap of the association between the different clustering subgroups and the clinicopathological parameters of STAD patients.

### 3.6. Construction and Validation of a Novel Signature m6ASig Based on DEGs

**Figure 5:**
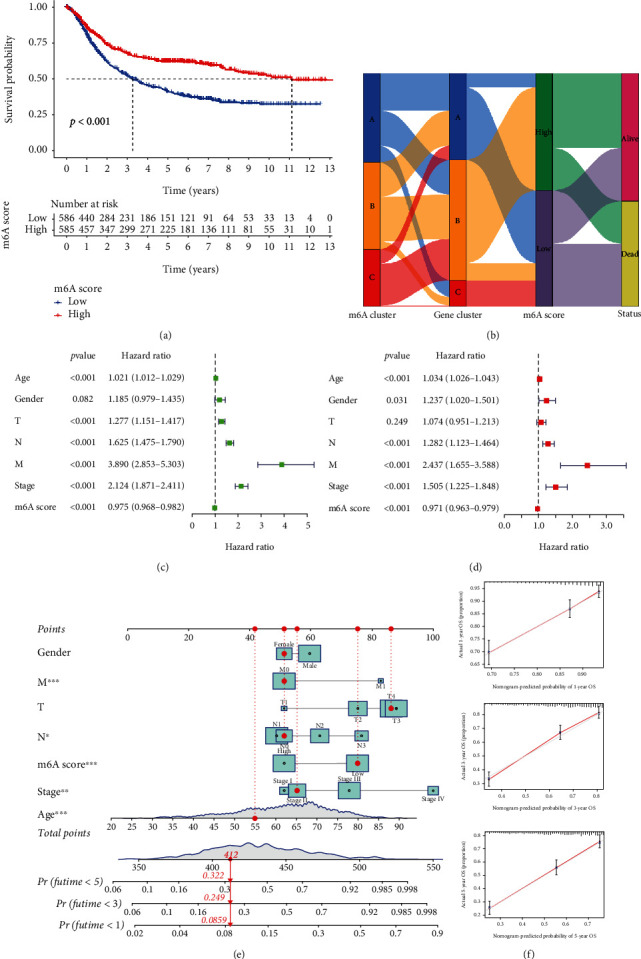
Construction of the m6ASig scoring system and its clinical prognosis analysis in STAD patients. (a) Kaplan-Meier curves for high and low m6ASig score STAD patient groups in TCGA-GEO metacohort (log-rank test, *P* < 0.001). (b) Sankey plot for the change of patients in different subgroups. (c) Univariate cox regression analysis for the m6ASig scoring model. (d) Multivariate cox regression analysis for the m6ASig scoring model. (e) A nomogram performed based on the results of cox regression analyses. (f) Calibration plots for predicted and actual 1-, 3-, and 5-year overall survival probability comparison.

To further quantify the m6A modification patterns of each STAD patient, a scoring system, named as m6ASig, was constructed. STAD patients were separated into two groups according to the m6ASig: the high-m6ASig score group (*n* = 585) and low-m6ASig score group (*n* = 586) (Table S4). K-M curves showed that patients in the high-m6ASig score group had a notably better OS than the low-m6ASig score group (Figure 5(a), *P* < 0.001). The Sankey map depicted that patients with high m6ASig were mainly connected with gene cluster B and showed better living status (Figure 5(b)). The univariate analysis revealed that the age (*P* < 0.001, HR = 1.021, 95% CI = 1.012‐1.029), T stage (*P* < 0.001, HR = 1.277, 95% CI = 1.151‐1.417), N stage (*P* < 0.001, HR = 1.625, 95% CI = 1.475‐1.790), M stage (*P* < 0.001, HR = 3.890, 95% CI = 2.853‐5.303), stage (*P* < 0.001, HR = 2.124, 95% CI = 1.871‐2.411), and m6ASig (*P* < 0.001, HR = 0.975, 95% CI = 0.968‐0.982) were significantly correlated with the OS (Figure 5(c)). Moreover, when all the parameters were enrolled in the multivariate cox regression model, the age (*P* < 0.001, HR = 1.034, 95% CI = 1.026‐1.043), N stage (*P* < 0.001, HR = 1.282, 95% CI = 1.123‐1.464), M stage (*P* < 0.001, HR = 2.437, 95% CI = 1.655‐3.588), stage (*P* < 0.001, HR = 1.505, 95% CI = 1.225‐1.848), and m6ASig (*P* < 0.001, HR = 0.971, 95% CI = 0.963‐0.979) were identified as independent prognostic predictors (Figure 5(d)). Patients with high m6ASig also had better OS in patients from different TNM stages (Figure S4).

Additionally, an independent dataset GSE15459 was applied to validate the prognostic role of the m6ASig model (Figure S5). Collectively, all the analyses demonstrated that patients in the high m6ASig group had better prognosis than the low score group. To further strengthen the predictive accuracy, a nomogram according to the results of cox regression analyses was established. As shown in Figure 5(e), higher total points were related to worse 1-year, 3-year, and 5-year OS. For instance, if a 55-year-old female patient with low m6ASig was diagnosed with T4N0M0 (IIB) STAD, she would get a total score of 412 points, which predicted the 1-year, 3-year, and 5-year OS rates of 91.4%, 75.1%, and 67.8%, respectively. The calibration curves illustrated excellent predictive performance compared with the ideal model of the 1-year, 3-year, and 5-year OS curves (Figure 5(f)).

### 3.7. Association between Different m6ASig Groups and Tumor Immune Landscape

**Figure 6:**
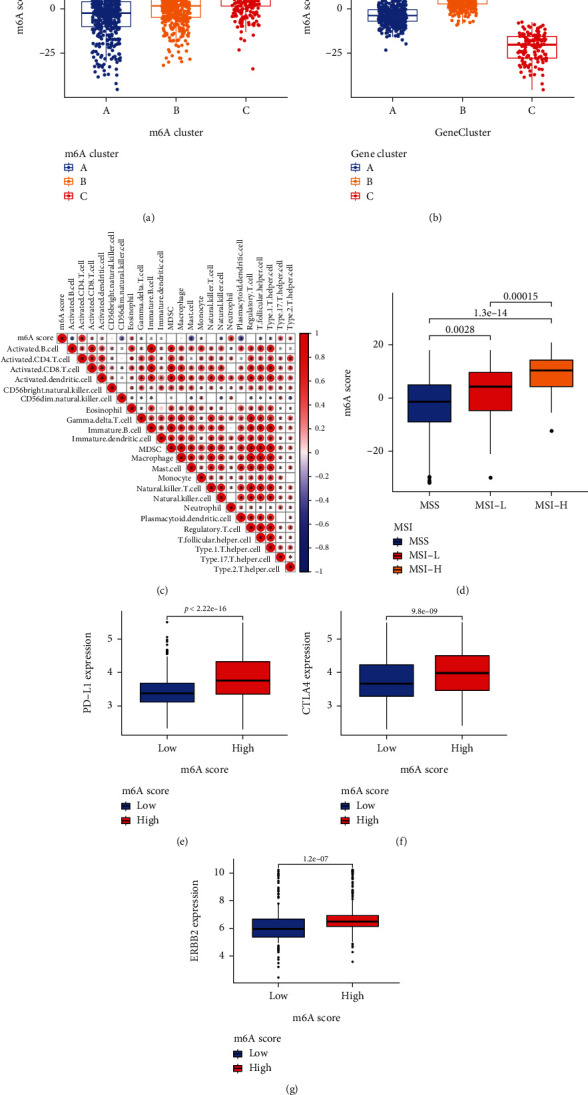
Associations of the tumor microenvironment with different m6ASig groups in STAD patients. (a) Differences in m6ASig score among three m6A clusters in TCGA-GEO metacohort (*P* < 0.001). (b) Differences in m6ASig score among three gene clusters in TCGA-GEO metacohort (*P* < 0.001). (c) Correlations between m6ASig score and the 23 immune cells. Negative correlation was marked with blue and positive correlation with red. (d) Correlations between m6ASig score and MSI status. MSS: microsatellite stable; MSI-H: high microsatellite instability; MSI-L: low microsatellite instability. (e–g) Expression of common drug targets among low and high m6A score groups. Drug targets including PD-L1 (e), CTLA4 (f), and ERBB2 (g).

To better illustrate the features of m6ASig scoring system, we further assessed the correlation between m6ASig score and m6A clusters/m6A gene clusters. Results demonstrated that there were statistically differences of m6ASig score level among different subtypes (Figures 6(a) and 6(b), all *P* < 0.001). The correlation between m6ASig score and 22 types of infiltrating immune cells was analyzed via Spearman correlation analysis. As shown in Figure 6(c), m6ASig score was most connected with activated CD4 T cell, while mast cell and plasmacytoid dendritic cell had a strong negative connection with m6ASig. Next, our study assessed the relationship between m6ASig score and MSI in the TCGA database. Results showed that there was a notably difference of m6ASig score level among patients with different MSI status (*P* < 0.01). Specifically, patients with MSI-H showed the highest m6ASig score (Figure 6(d)). In addition, we also found that patients with high m6ASig score had higher PD-L1, CTLA4, and ERBB2 expressions than patients with low score in the metacohort (Figures 6(e)–6(g), all *P* < 0.001).

**Figure 7:**
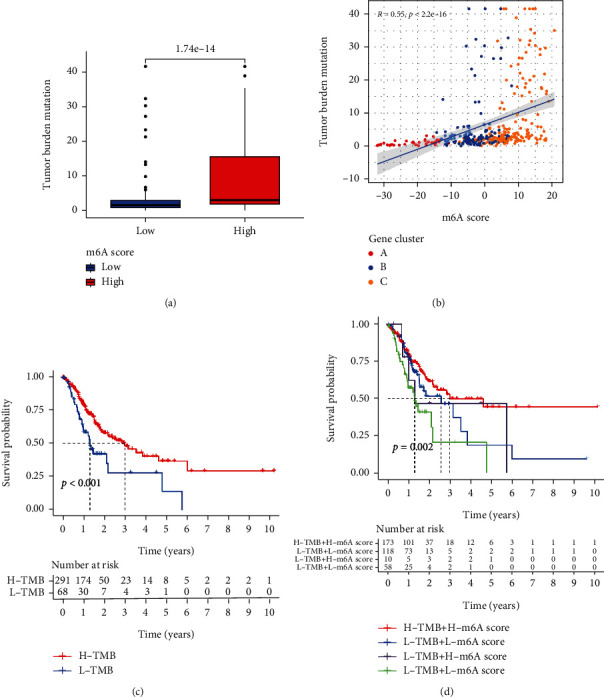
Associations of tumor mutation burden (TMB) with different m6ASig groups in STAD patients. (a) Stratified analysis of the m6ASig score for STAD patients by TMB (*P* < 0.001, Wilcoxon test). (b) A scatter plot of correlation analysis revealed a positive connection between TMB level and m6ASig score. (c) Kaplan-Meier curves for OS for high and low TMB groups (log-rank test, *P* < 0.001). (d) Kaplan-Meier curves for OS for the patients stratified by both the m6ASig score and TMB (log-rank test, *P* = 0.002).

The correlation between TMB and m6ASig was also explored in TCGA cohort. Patients with high m6ASig score exhibited higher TMB levels than those with low score (Figure 7(a), *P* < 0.001). The correlation analysis revealed that TMB level was positively associated with higher m6ASig score (Figure 7(b)). K-M survival analysis showed that H-TMB patients had longer OS (Figure 7(c), *P* < 0.001). What is more, combination of TMB and m6ASig in K-M analysis demonstrated that STAD patients with higher TMB and m6ASig score had the most satisfying outcome (Figure 7(d), *P* = 0.002).

### 3.8. Clinical Application of m6ASig Model for Chemotherapy and Immunotherapy Response Prediction

**Figure 8:**
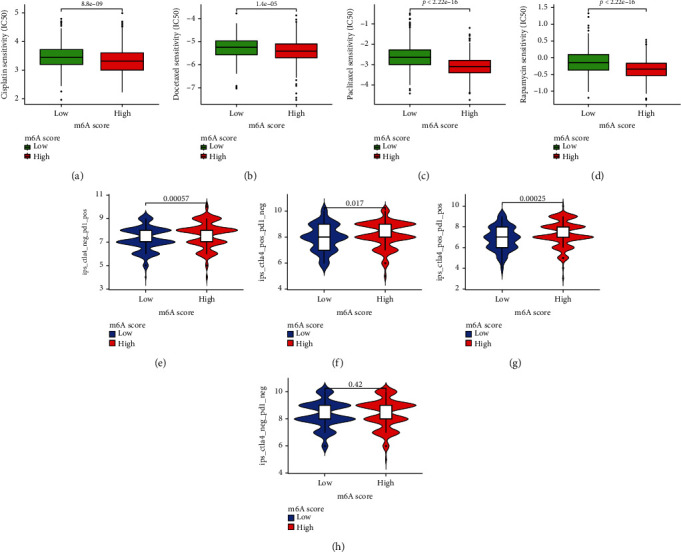
Prediction of immunotherapy and chemotherapy response. (a–d) Sensitivity analysis of four common chemotherapeutic drugs in STAD patients with high or low m6ASig scores. Drugs including cisplatin (a), docetaxel (b), paclitaxel (c), and rapamycin (d). (e–h) The relative distribution of immunophenoscore (IPS) between high or low m6ASig score groups. *P* < 0.05 was considered significant.

Then, we went further to assess the clinical value of our m6ASig scoring model in chemotherapy and immunotherapy response prediction. IC_50_ was used to predict the drug sensitivity of four common drugs for treatment of STAD: cisplatin, docetaxel, paclitaxel, and rapamycin. We found that patients in the high-m6ASig score group were more sensitive to all of the 4 drugs compared with the low score group (Figures 8(a)–8(d), all *P* < 0.001). Subsequently, the association between IPS and m6ASig score in immunotherapy was assessed. Results showed that high-m6ASig groups had higher IPS scores in the IPS-CTLA-NEG-PD1-POS, IPS-CTLA-POS-PD1-NEG, and IPS-CTLA-POS-PD1-POS groups (Figures 8(e)–8(g), *P* < 0.001, *P* = 0.017, and *P* < 0.001, respectively), which indicated that high m6ASig score patients had better opportunity to benefit from immunotherapy.

## 4. Discussion

The TCGA project was launched in 2005 to improve our understanding on the genetic diversity of different cancers via innovative genome analysis methods, assisting clinicians to generate new ideals for cancer treatment [27]. Here, in our study, using multiple bioinformatics algorithms, we identified three distinct m6A regulators and m6A-related gene clusters which were correlated tightly with STAD patients' survival and TME immune cell infiltration. Moreover, we also established a scoring system: m6ASig to associate m6A mediation patterns with tumor immune landscape and predict immunotherapeutic and chemotherapy efficacy on each STAD patients.

Firstly, our study assessed the RNA expression and genetic mutations of 23 m6A regulators. Our prognostic analyses demonstrated that YTHDF1, IGFBP1, IGFBP2, IGFBP3, FTO, and ALKBH5 were unfavorable factors for STAD patients' OS while patients with higher levels of WTAP, RBM15, YTHDC2, TYHDF2, YTHDF3, HNRNPC, and LRPPRC were connected with better OS. Among which, YTHDF1 [8], IGFBP1 [28], IGFBP2 [29], FTO [30], ALKBH5 [31], WTAP [32], YTHDC2 [33], and LRPPRC [34] have been previously reported to predict the prognosis of STAD patients. For instance, YTHDF2 could induce degradation of the transcripts by selectively recognizing and binding to m6A sites [35]. Shen et al. previously reported that overexpression of YTHDF2 could inhibit tumor proliferation in gastric cancer through regulating FOXC2 signaling pathway and may serve as a favorable prognostic factor [36]. In our prognostic analysis, patients with higher levels of YTHDF2 were also connected with better OS. Based on 17 m6A regulators, we built three distinct m6A regulator clusters. These three clusters had significantly distinct survival outcomes and immune cell infiltration levels. Cluster C had the best OS compared with the other two clusters and could be characterized as an immune-inflamed phenotype [37], with the presence of activated CD4 T cells, activated dendritic cells, gamma delta T cells, and natural killer cells. However, though cluster B had a relatively rich abundance in tumor-infiltrating immune cells, including B cells, and MDSC, the patients in cluster B did not show survival benefit compared with other clusters. This phenomenon may be explained that those immune cells are restricted in the stroma surrounding the tumors but are not penetrated into the tumors, thus making the antitumor activity ineffective [37]. Furthermore, in GSEA analysis, compared with cluster B, cluster C was mainly involved in base excision repair and homologous recombination pathways.

Next, our study identified 229 m6A hallmark genes from the three m6A clusters. These differentially expressed genes were considered as products of m6A regulators' posttranscriptional modifications. Just alike to the results of m6A regulator clustering, the three gene clusters could distinguish STAD patients with different clinical outcomes. GO and KEGG enrichment analyses demonstrated that those hallmark genes were mainly involved in the signal pathways related to cancer development and metastasis, such as transcriptional misregulation and Wnt signaling pathway. Among the 229 m6A hallmark genes, some of them had been well studied in STAD. For instance, RIPK2 had been previously reported to play an important role in modulating gastric cancer (GC) cell proliferation, migration, and apoptosis through the NF-*κ*B signaling pathway [38]. A recent study found that DHRS3 was hypermethylated and downregulated in GC patients and reduced expression of DHRS3 is implicated in gastric carcinogenesis, which suggested DHRS3 could be a tumor suppressor [39]. However, the exact biological functions of most hallmark genes in STAD patients have not been well explored which needed further evaluation in future studies.

To further quantify the m6A modification patterns in individual patients, we constructed m6ASig scoring system based on the gene clusters, aiming to predict the survival and therapeutic strategies for each STAD patients more accurately. Our results demonstrated that the m6A cluster C, which was characterized as immune-inflamed phenotype, had higher scores of m6ASig. Survival analysis further validated that high m6ASig score was correlated with better OS. Our study also used a nomogram according to the results of cox regression analyses to improve the accuracy of prognostic prediction and help clinical decision-making. The prognostic role of the m6ASig model was further validated in an independent dataset GES15459. Moreover, our study found that higher m6ASig score was positively associated higher levels of TME immune cells, such as activated CT4 cells, CD8 cells, dendritic cells, gamma delta T cells, type 2 and type 17 T helper cells, and neutrophils. All these results indicated that our m6ASig was a reliable scoring system for the comprehensive analysis of m6A modification on individual STAD patients and could predict the level of immunotherapy response by characterizing the TME immune cells.

Immunotherapy is arising as a powerful clinical strategy for cancer treatment. However, only a limited number of patients could benefit from current immunotherapies. Clinical trials revealed that MSI-H patients have better prognosis than microsatellite stable patients, owning to the activation of T lymphocyte recognition of neoantigens [40, 41]. In consistent with previous researches, we found that patients with high m6ASig score exhibited higher MSI-H status. Other well-recognized immune response predictors, such as PD-L1, CTLA4, and ERBB2, were all positively connected with m6ASig score. High TMB is linked to prolonged survival in various cancers [42–44]. Our study found that patients with higher m6ASig score exhibited higher TMB level and STAD patients with higher TMB and m6ASig score had the most satisfying survival compared with other groups. Immunophenoscore (IPS) was used to assess response to immune checkpoint inhibitors. Results showed that high m6ASig score group had higher IPS scores in the IPS-CTLA-NEG-PD1-POS, IPS-CTLA-POS-PD1-NEG, and IPS-CTLA-POS-PD1-POS groups, which indicated that those treatments were more efficacious in patients with high m6ASig scores. Besides immunotherapy response prediction, our study also evaluated the clinical use of our m6ASig model in chemotherapy response. We found that patients in the high m6ASig score group were more sensitive to the common drugs for STAD treatment. All these findings suggested that our m6ASig could not only predict STAD patients' clinical responses to immunotherapy but also the efficiency of chemotherapy.

Several recent studies on m6A modification have outlined its key role in STAD patients. Zhang et al. were the first to reveal that the m6A modification played a nonnegligible role in formation of TME diversity and complexity [45]. In a recent study, Meijing et al. reported that STAD patients in the high m6A score group had a better prognosis than those in the low m6A score group [46], which were similar with our results. However, there are still many differences between the two articles. First of all, the sample size of Meijing et al.'s study was relatively small (TCGA + GSE84437, total patient number: 876), while our study included 1246 STAD patients. It was known that bigger sample size might present more solid results. Secondly, after construction of the m6A scoring system, our study further used a predictive nomogram by integrating different prognostic factors to further strengthen the predictive accuracy. The calibration curves illustrated excellent predictive performance compared with the ideal model of the 1-year, 3-year, and 5-year OS curves. Thirdly, our study assessed the clinical value of our m6ASig scoring model both in chemotherapy and immunotherapy response prediction. However, Meijing et al. did not conduct drug sensitivity analysis, thus making their m6A scoring system lack of information for chemotherapy response prediction. Last but not least, we further selected GSE15459 dataset as an external validation cohort to confirm the prognostic value of m6A regulators in gastric cancer. Results demonstrated that the external validation cohort presented the consistent results thus confirming our findings.

However, several limitations should be noticed in our study. First, TCGA and GEO cohorts only shared the information of 17 m6A regulators; 6 of the 23 regulators were excluded from our study. Second, since our study was a retrospective study based on bioinformatics analysis, future prospective researches are needed to validate our findings, and mechanism researches at cellular and molecular levels are needed to fully elucidate the exact function of m6A regulators and hallmark genes.

In conclusion, our study comprehensively evaluated the m6A modification patterns among STAD patients and established an m6ASig scoring system that could characterize TME immune cell infiltration, thus predict patient's prognosis and immunotherapy and chemotherapy efficacy, offering a novel tool for the individualized therapeutic implications for STAD patients.

## Figures and Tables

**Table 1:** Demographic and clinical characteristics of patients (*P* value: the result of chi-squared test).

	TCGA	GSE57303	GSE62254	GSE84437	Total
Number	443	70	300	433	1246
Age					
≤60	142	29	117	194	482
>60	296	39	183	239	757
Unknown	5	2	0	0	7
Gender					
Male	285	52	199	296	832
Female	158	18	101	137	414
T					
T1	23	0	0	11	34
T2	93	7	186	38	324
T3	198	54	91	92	435
T4	119	9	21	292	441
Unknown	10	0	2	0	12
N					
N0	132	13	38	80	263
N1	119	26	131	188	464
N2	85	26	80	132	323
N3	88	5	51	33	177
Unknown	19	0	0	0	19
M					
M0	391	63	273	433	1160
M1	30	7	27	0	64
Unknown	22	0	0	0	22
TNM stage					
I	59	3	30	21	113
II	130	9	96	138	373
III	183	41	95	274	593
IV	44	17	77	0	138
Unknown	27	0	2	0	29

## Data Availability

The data that support the findings of this study are available from the corresponding authors upon reasonable request.

## Supplementary Materials

Supplementary Materials Figure S1: flow chart of the study design. Figure S2: results of gene set variation analysis (GSVA) for the three m6A clusters. (A) Results of GSVA analysis for cluster A. (B) Results of GSVA analysis for cluster B. (C) Results of GSVA analysis for cluster C. Figure S3: cluster and functional analyses of m6A-related genes. (A) Venn diagram showing the number of DEGs among the three m6A clusters. (B) KEGG analysis of the common differential expressed genes. (C) GO enrichment analysis of the common differential expressed genes. Figure S4: Kaplan-Meier curves for high and low m6ASig score patient groups in the TCGA-GEO metacohort (log-rank test, *P* < 0.001). (A) STAD patients with TNM stage I-II (log-rank test, *P* < 0.001). (B) STAD patients with TNM stages III-IV (log-rank test, *P* < 0.001). Figure S5: validation of the m6ASig scoring model in GES15459. (A) The overlap among clusters when *k* = 3. (B) Principal component analysis (PCA) of m6A hallmark genes. (C) Kaplan-Meier curves for high and low m6ASig score patients (log-rank test, *P* = 0.04). Table S1: demographic and clinical characteristics of patients in GSE15459. Table S2: m6A cluster different genes. Table S3: m6A cluster-related hallmark genes. Table S4: patients' information in m6ASig score groups.
