# Supplementary material for: Integrated Analyses of m6A Regulator-Based Signature on Its Clinical Application and Immunogenomic Landscape in Stomach Adenocarcinoma
Source: Biomed Res Int. 2022 Sep 20;2022:2053719. doi: 10.1155/2022/2053719 (PMC9526603; doi:10.1155/2022/2053719)
Supplement: Supplementary Materials — Figure S1: flow chart of the study design. Figure S2: results of gene set variation analysis (GSVA) for the three m6A clusters. (A) Results of GSVA analysis for cluster A. (B) Results of GSVA analysis for cluster B. (C) Results of GSVA analysis for cluster C. Figure S3: cluster and functional analyses of m6A-related genes. (A) Venn diagram showing the number of DEGs among the three m6A clusters. (B) KEGG analysis of the common differential expressed genes. (C) GO enrichment analysis of the common differential expressed genes. Figure S4: Kaplan-Meier curves for high and low m6ASig score patient groups in the TCGA-GEO metacohort (log-rank test, P < 0.001). (A) STAD patients with TNM stage I-II (log-rank test, P < 0.001). (B) STAD patients with TNM stages III-IV (log-rank test, P < 0.001). Figure S5: validation of the m6ASig scoring model in GES15459. (A) The overlap among clusters when k = 3. (B) Principal component analysis (PCA) of m6A hallmark genes. (C) Kaplan-Meier curves for high and low m6ASig score patients (log-rank test, P = 0.04). Table S1: demographic and clinical characteristics of patients in GSE15459. Table S2: m6A cluster different genes. Table S3: m6A cluster-related hallmark genes. Table S4: patients' information in m6ASig score groups. [file 2053719.f1.docx]

**Please double click the icon, thank you!**
